# Smoke-free homes: what can we learn from adopting patient and public involvement in clinical practice in two countries to reduce exposure to secondhand smoke?

**DOI:** 10.1177/17579139251317845

**Published:** 2025-05-13

**Authors:** K Frazer, N Bhardwaj, C Bell, A Lyons, N Vickers, L Geoghegan, T McDonnell, D McGillicuddy, P Fitzpatrick, C Kelleher, T Kroll

**Affiliations:** University College Dublin, Health Sciences Centre, Dublin, 4, Ireland; University College Dublin, Ireland; Southern Health Board NI, UK; St Vincent’s University Hospital, Ireland; University College Dublin, Ireland; Northern-Ireland Department of Health, Social Services and Public Safety, UK; University College Dublin, Ireland; University College Dublin, Ireland; University College Dublin, Ireland; University College Dublin, Ireland; University College Dublin, Ireland

## Background

Tobacco use is acknowledged as the primary preventable cause of death globally, and the World Health Organization (WHO) European Region estimates over 8.7 million deaths per year, including approximately 1.3 million in people who do not smoke and are exposed to secondhand smoke (SHS).^1–4^ The harmful effects are increased in enclosed spaces.^5^ It is 20 years since the International Agency for Research on Cancer published evidence linking tobacco smoke and involuntary (passive) smoking to several cancers.^6^ We know there is no safe level of SHS, and exposure is associated with heart disease and cancer, among other acute and chronic diseases. In pregnancy and postnatally, exposure increases the risk of premature birth, low birth weight, and sudden infant death syndrome. Thus, a majority of SHS exposures occur in homes.^4,7,8^

Article 8 of the WHO Framework Convention on Tobacco Control (FCTC)^9^ supports countries in protecting populations from exposure to SHS, and currently, 182 countries are parties to the treaty.^2^ In the 20 intervening years, we acknowledge that comprehensive legislation protecting people from exposure to SHS has increased from 5% globally in 2008 to 25% in 2022.^4^ Despite these improvements, it is evident that global challenges persist^1,10^ to achieve the human right to clean air and to achieve the Sustainable Development Goal targets.^11,12^

Tobacco control is critical in achieving worldwide success for the Sustainable Development Goals (SDGs). The WHO concedes slow progress and seeks widening access to cessation support. Developments are urgent, with evidence of increased rates of smoking at home during the COVID-19 pandemic, thereby increasing risks for children in greatest need.^13^

To understand more about reducing smoking in the home, the Economic Social Research Council and Irish Research Council, in collaboration with the Economic Research Council (UK), funded a network award to establish a consortium comprising five countries to consider SHS and share knowledge and expertise. Patient and public involvement (PPI) must be at the heart of planning and developing services, shaping innovative and realistic solutions in the Irish context and is embedded in the national funding agency strategy.^14^ As part of a series of activities during the funding award (2021/2022), an online workshop was planned to consider key questions to support people wanting to quit smoking and reduce smoking in the home.

## Methods

A virtual workshop was completed in January 2022 to commence the development of a PPI engagement process to understand how to support reducing SHS exposure in homes. The workshop was held virtually due to the impact of the COVID-19 pandemic during this time (2021/2022).

## Aim

The aim was to identify *fit-for-purpose solutions* to address the challenges at-risk populations face in reducing SHS exposure in homes.

## Participants

Those invited to participate were practitioners engaged in smoking cessation activities in several settings in two of the consortium’s countries: Northern Ireland and Ireland. The participants’ smoking cessation expertise included community-based practice, general hospitals, and maternity services. Other participants invited were researchers, academics, and people working in the third sector (charitable organisations) who support at-risk groups. People who had previously quit smoking were invited via third-party invitations.

## Ethics

A university human ethics committee (LS-E-21-181) granted low-risk ethical approval for the public engagement activity in July 2021. The virtual workshop was not recorded, and no demographic data were sought.

## Data Collection

In total, 20 people were invited to participate in a 2h online facilitated workshop, and 14 attended. Attendees comprised several smoking cessation practitioners representing Northern Ireland and Irish health systems, academics, researchers, third-sector organisations and members of the public.

Following introductions and setting the scene for the day, participants were divided into breakout rooms to facilitate engagement and discuss specific questions to guide the session. Feedback from the three rounds of discussions was captured via online digital whiteboards to support open communication and anonymity. The questions posed in three separate rounds in breakout rooms are shown in Table 1.

**Table 1:** Questions for each workshop activity.

Round 1	Round 2	Round 3
What are the challenges to creating environments to support behaviour change and reduce secondhand smoke exposure for others?	Who is Invisible to services? What would need to happen for their engagement? Who is missing from your services, yet is a high-risk group exposed to secondhand smoke?	Looking at our solutions from round 2- what options would you fund, and by which amount (€1000, €10,000, €100,000)?
Who are the key at-risk groups, and where are they located?	Who are Visible and have yet to engage with services? Under involved in services. What would need to happen for their engagement (solutions)?	What do you need for the solution to start?
What is unique about their situation that the challenge continues for them?	Who are Engaged in tobacco control services, and it has yet to work? What needs to change to support this group (solutions)?	What can you do to make this happen?

## Analysis

After the meeting, a draft summary document of notes compiled during the meeting, documented information, and notes from breakout room conversations was shared with all attendees for member checks and clarification. Feedback was incorporated into a final draft, and permission was sought for inclusion as named co-authors. Examples of the Jamboards are provided for transparent reporting (Photos 1, 2 and 3).

## Photos 1 to 3 Examples of Responses Obtained from Participants


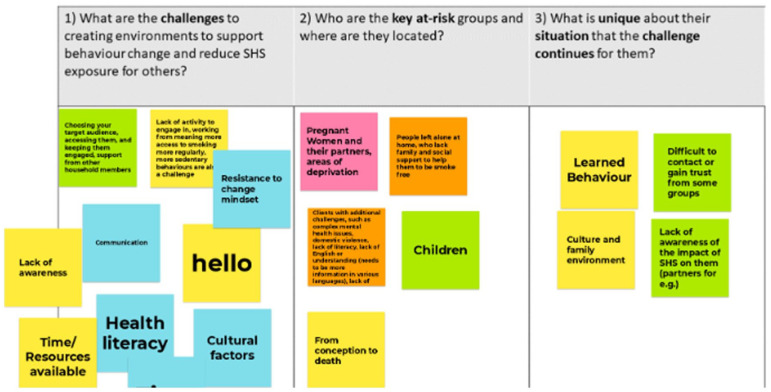



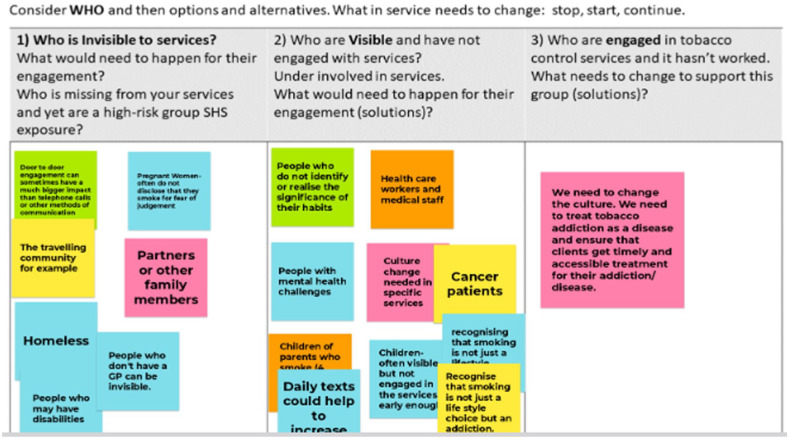



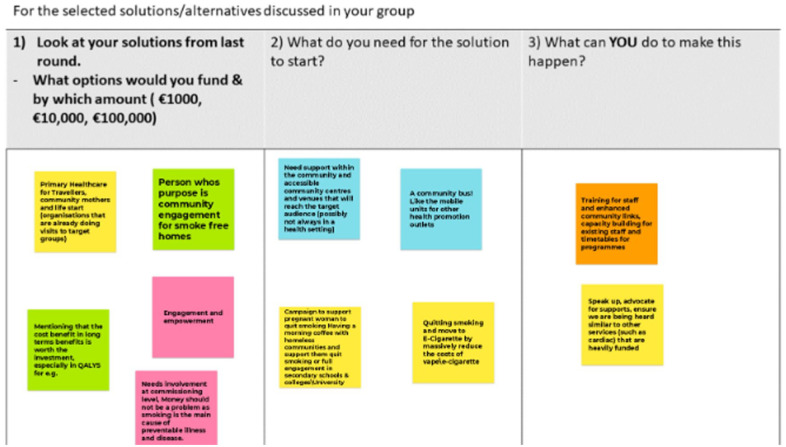


## Results

### Round 1

**Figure 1:**
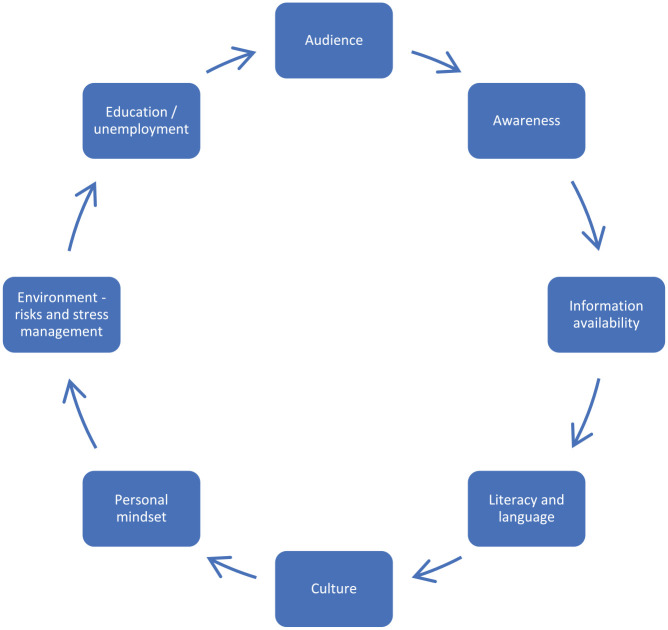
What creates and supports exposure to secondhand smoke in homes

The conversations within the three breakout rooms identified several challenges in creating environments to reduce exposure to SHS and in enabling people to have the capability to engage in behavioural change. Figure 1 provides an overview of the discussions.

Participants identified several factors that increase the risks of and continued exposure to SHS in homes. The impact of the wider social and structural determinants of health was evident. However, limitations may exist in understanding the harms of SHS exposure due to the presentation of health information for those with literacy difficulties and the limited availability of resources in languages other than English.

Contributing factors that could influence a healthcare professional to discuss quitting included an acknowledgement that home life and living spaces vary for individuals and may consist of high-stress environments, including homes where people experienced or were at risk of domestic violence – where women may feel unsafe discussing their risk of SHS exposure with a partner, for example. Other situations noted during the discussions include people experiencing emotional difficulties due to COVID-19 with reduced opportunities for engaging in alternative supports. Other key factors that could impact discussing quitting smoking include people who are unemployed and those living in challenging environments, including precarious home environments and/or experiencing homelessness, with limited resources. Personal difficulties and normalised coping mechanisms used by people who smoke may have resulted in increased consumption during the COVID-19 pandemic (the timeline of data collection). Additional wider determinants of health include a lack of awareness of SHS, culture and social norms around smoking behaviours and smoking in the home, and many people being unaware of the risks. The timing of conversations about risks and the information that should be communicated were critical issues.

In healthcare settings, conversations about quitting may not include a family member or another person, and for people who are not connected to a GP or other services, they may not exist in the healthcare system, that is, they are invisible. Children are unaware of their risks and depend on adults to make decisions and provide a safe environment. Children may be engaged with services, including healthcare (attending hospitals or clinics, for example), and no conversations are included relating to quitting or supporting quit attempts with a parent or carer. It may be similar for carers or carers in the home – no data are recorded about the wider context of the environment in which they live. For the person who smokes, there may be a lack of awareness of the risks posed to anyone beyond themselves, and they may not have engaged with any literature or sought advice on this topic.

At-risk groups identified during the workshop included pregnant women and their partners, children, and people who live alone with limited support to engage in assisting a quit attempt. The risks were acknowledged to be present from conception to death, in that there is no safe exposure to SHS. The challenges for the at-risk groups and their situations included learning behaviours, the impact of wider social determinants of culture and family environment, and a lack of awareness of the impact of SHS on partners, for example. The view of participants overall was that the message of SHS exposure in homes and the associated risks is not widely known or understood in ways that people can comprehend regarding their circumstances.

### Round 2

**Figure 2:**
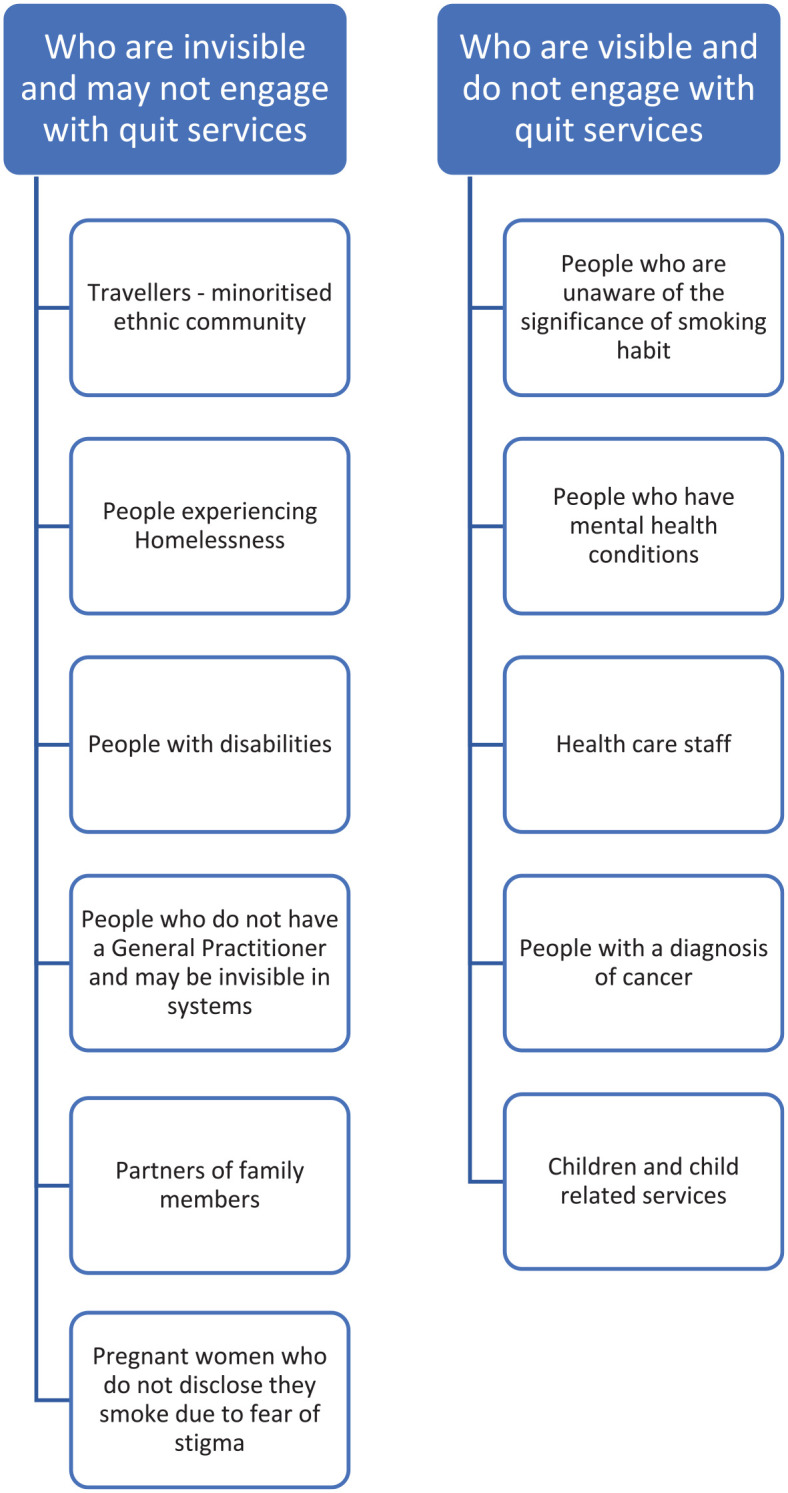
Engagement with quit services and visibility

In this round, participants were asked to consider the ‘who’ in relation to who accesses quit services and who does not. What changes are necessary and should be considered under stop, start and continue? Figure 2 identifies the key groups identified by participants, for those who are invisible and are visible and attending or engaged with quit services. One of the solutions identified to support engagement with smoking cessation services was a culture change – to treat tobacco addiction as a disease – and not see it solely as a behavioural change and a personal issue; smoking is not a lifestyle choice and is an addiction. This reframing was supported by participants in all group discussions.

### Round 3

**Figure 3:**
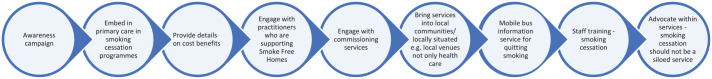
Solutions to support engaged development

Finally, in round 3, the participants identified several wide-ranging solutions for improving services. While participants did not assign monetary values to their solutions (due to time constraints), the suggestions outlined in Figure 3 provide data to develop further opportunities and engagement. Greater awareness to improve communication of SHS was noted and linked to other methods used in prevention campaigns, including the use of mobile buses for information on tobacco control and SHS. What is interesting is the range of opportunities noted for embedding tobacco control and conversations on SHS in current services – to co-exist. The separation and siloed tobacco control services currently do not reach all of the at-risk groups. Suggestions noted embedding services in existing primary care programmes and bringing engagement to locations that are not healthcare settings. The importance of community focus emerged both as a location and to enhance capacity building. Increasing service provision solutions included commissioning more services at the community level and advocating to expand existing hospital-based services, which participants considered ‘well-funded’ due to consistent and regular staff training.

## ‘Bringing it all Together’

The workshop concluded with agreement on the positive impact of the engagement and the welcomed opportunity of space for sharing similar problems and considering opportunities for networking and collaboration. This forum does not exist. The voices of those who were not healthcare practitioners, those who represented groups at risk and those who had previously smoked were critical for learning at the PPI workshop.

The evidence from this PPI-led facilitated workshop identified opportunities to co-develop resources to understand SHS exposure and work with many of the communities and groups, as noted in Figure 3. It is evident from the conversations that the key role of health literacy is developing and communicating health messages that are tailored for community involvement. Changing behaviour has wider implications, and it is critical to understand the capabilities, opportunities and motivating factors that exist in a person’s life and the wider impact of social determinants of health.^15–17^

Since this workshop was completed, Slaintecare in Ireland commenced a Healthy Communities programme in pilot areas *to provide increased health and wellbeing services in 19 community areas across Ireland*. This initiative includes increased access to smoking cessation programmes and nicotine replacement therapies.^18^ Tobacco Free Ireland continues to expand the services provision in communities with marginalised communities,^19^ and this is welcomed, given the challenge that smoking rates have stayed the same since 2019.^20^ A recent report and review of tobacco control in Northern Ireland report higher smoking rates in Ireland and Northern Ireland when compared to Scotland and Wales, with the highest rates noted in marginalised communities and confirming the challenge of reduced funding for tobacco control media campaigns between 2020 and 2024.^21^

The PPI-led workshop presents opportunities to develop a whole-of-Island approach to reducing exposure to SHS in Ireland and Northern Ireland and discuss how to support those at greater risk in homes. Improving communication methods is key across the two countries regarding the risks of SHS exposure, supporting health literacy for groups at continued risk, and developing mechanisms to improve general awareness, especially for and with those at the highest risk, so that no one is left behind. These opportunities remain at the heart of the Ottawa Charter^22^ to create supportive environments and promote the health and wellbeing of communities and societies. The relevance of the Ottawa Charter persists almost four decades later.^23–26^ It can be achieved by working with high-risk groups to co-develop and co-design realistic supports and tailored approaches.

## Limitations

There are several limitations associated with this study. The views and narrative represent those who attended, which may exclude broader opinions. The participants who attended and contributed to the workshop represented clinical practice, smoking cessation services, academics, and members of the public. The workshop was held online, and as a result, representation of marginalised groups is limited. We acknowledge the input of those who attended and appreciate that the platform used may not have encouraged others. The data reported were not recorded, and no demographic data were sought. While the data would be helpful to present specifics within this article, we acknowledge that the ethical approval sought did not permit personal data collection, nor did we seek barriers to engagement. It may be that fewer people would have attended if the workshop had been recorded and personal data had been obtained.

## Conclusions

The voices of those who represented groups at risk and contributed their experiences of smoking and supporting those who wanted to quit were critical in providing essential and valued expertise in the PPI workshop. Overwhelmingly, the community as a setting was described by groups for capacity building. While the Healthy Communities programme and increased funding for prevention and quit services are acknowledged, there remains a gap for marginalised and at-risk groups. Ongoing training is essential for those with co-existing morbidities, as a one-size-fits-all approach is unsuitable. Structural barriers that contribute to the absence of engagement include a general lack of awareness of the impact of SHS exposure, which exists with limited communication of SHS messages and for at-risk populations. The language used for SHS, acknowledging a need for improved health literacy, should be prioritised to empower the key groups and inform policymakers.
